# Demographic and Phenotypic Distribution of Inflammatory Bowel Disease in 1015 Patients Attending a Quaternary Care Center in Nepal

**DOI:** 10.7759/cureus.83226

**Published:** 2025-04-29

**Authors:** Umid K Shrestha, Rabin Sharma

**Affiliations:** 1 Department of Gastroenterology and Hepatology, Nepal Mediciti Hospital, Lalitpur, NPL

**Keywords:** age, crohn’s disease, demographic, distribution, ethnic, gender, inflammatory bowel disease, nepal, phenotypic, ulcerative colitis

## Abstract

Objective

Inflammatory bowel disease (IBD), comprising ulcerative colitis (UC) and Crohn’s disease (CD), has been seen as an emerging health problem in Nepal with urbanization and the adoption of a Western lifestyle. Our study aimed to determine the various demographic and phenotypic features of IBD in Nepal.

Methods

This prospective observational study was conducted at the Nepal Mediciti Hospital (Lalitpur, NPL), comprising IBD patients from 2017 to 2024. The demographic distribution of IBD was classified according to age, gender, and ethnicity, while the phenotypic distribution was done according to disease location and severity of the disease.

Results

Among 1015 IBD patients, 752 (74.1%) had UC and 263 (25.9%) had CD. The IBD was predominantly seen in males (60.7% vs 39.3 %). The mean age at diagnosis of IBD was 39.67 ± 14.53 years, 38.3 ± 8.4 years for UC, and 31.8 ± 6.7 years for CD. Inflammatory bowel disease was more common (51.1%) in the Khas-Arya race, followed by Newars (18.8%). The most common location of UC was left-sided (43.8%), and ileocolonic for CD (55.5%). The majority of the IBD patients presented in a state of moderate activity (UC 50.1% and CD 62.4%), followed by mild activity (UC 39.2% and CD 29.3%). Only a few IBD patients presented in severe form (UC 10.6% and CD 8.4%).

Conclusion

This study highlights the increasing burden of IBD in Nepal, affecting more men than women, occurring at the mean age of thirties, and mostly seen in the Khas-Arya race. We found mostly left-sided disease in UC patients, while ileocolonic disease was the most common phenotype in CD. The majority of IBD patients in our study presented with moderate activity. Further large-scale, long-term, multi-center studies are needed to provide a more comprehensive knowledge of the clinico-epidemiological evaluation of IBD in Nepal.

## Introduction

Inflammatory bowel disease (IBD) is an idiopathic chronic intestinal disorder, which includes ulcerative colitis (UC) and Crohn's disease (CD) [1]. The underlying etiology and pathogenesis of IBD remain unclear, but are thought to result from an interaction between genetic susceptibility, environmental factors, and the host immune response [2]. Ulcerative colitis involves the colon in a continuous manner and almost always involves the rectum. On the other hand, CD can involve any part of the gastrointestinal (GI) tract from the mouth to the anus, though not in continuous pattern. Inflammatory bowel disease has been found mostly in Western populations. High prevalence rates have been reported from North America, United Kingdom, and Northern Europe [3,4]. However an increasing number of recent studies have suggested rising incidence and prevalence of IBD in Asia [5,6]. The reasons could be multi-factorial, and may at least partly be related to development and prosperity, resulting in improved hygiene and changing dietary habits [7]. 

Investigating the early stages of IBD as it emerges in new populations may provide a new opportunity to study its pathophysiology. Unfortunately, data on the epidemiological and clinical profile of IBD in many Asian countries, and most developing countries, are scarce [8]. The epidemiological and clinical information on IBD in many of these countries suffer from poor reporting networks, limited population-based data, hospital data from undefined catchment areas, and non-uniform diagnostic criteria.

Nepal as a country is going through economic transition with changes in disease patterns; there has been a gradual decline in diseases associated with poverty and an increase in non-communicable diseases. The IBD has been seen as an emerging health problem related to idiopathic chronic intestinal disorder in Nepal with urbanization and adoption of western lifestyle. In Nepal the IBD sub-type CD used to be thought as of a rare disease, as published in one article from Eastern Nepal in 2009 as a rare disease case report, but the author had raised a question whether it was a true rarity or a result of gross under-diagnosis [9]. With the change in the disease pattern and improvement in the diagnostic modalities, the increasing numbers of IBD, both UC and CD, are being identified in Nepal over the last decade. Despite the increasing cases of IBD, there are very few published studies about IBD in Nepal till date [10-15], and a large sample study of IBD is still lacking. Hence, the aim of our study was to determine the various demographic and phenotypic features of IBD in Nepal.

## Materials and methods

This is a prospective observational study with a total of 1015 new IBD patients whose details were collected from November 2017 to October 2024 (a total of seven years) at Nepal Mediciti Hospital (Lalitpur, NPL), a quaternary care center of Nepal. This study was approved by the Institutional Review Committee (IRC) of Nepal Mediciti Hospital (approval no. IRC-CR-2074/75-0001). Care was taken not to overlap patients with IBD. 

Patients with IBD were categorized into UC and CD. The demographic distribution of IBD was classified according to age, gender, and ethnicity. The ethnic group of Nepal was grouped into Khas-Arya, Janajatis, Newars, Madhesi, and others, as per the census of Nepal [16]. The exclusion criteria for the study were patients ≤ 10 years of age, indeterminate colitis, and infective colitis. Diagnosis of IBD was made by conventional clinical, radiological, colonoscopic, and histological criteria [17]. The upper GI endoscopy was not routinely performed in our patients unless they had symptoms. 

The phenotypic classification of IBD was done to categorize IBD into subtypes based on disease location and severity of the disease. According to disease location, patients with UC were classified per the Montreal classification, i.e., E1: ulcerative proctitis (proximal extent of inflammation distal to the rectosigmoid junction), E2: left-sided UC (distal UC: involvement limited to the portion of the colorectum distal to the splenic flexure), and E3: extensive UC (pancolitis: involvement extended proximal to the splenic flexure) [18]. The severity of UC was determined according to the Truelove and Witts criteria [19]. The CD patients were characterized by disease location and activity. The disease location was determined according to the Montreal classification (L1: terminal ileum, L2: colon, L3: ileocolon, L4: isolated upper gastrointestinal disease) [18], and the clinical activity was measured per the Crohns’s Disease Activity Index (CDAI), namely mild, moderate and severe [20]. The behavior of CD was not available for all patients; hence, it was not considered during the study.

Statistical analysis was done using SPSS Statistics version 27.0.1 (IBM Corp., Armonk, NY, USA). Continuous data were expressed as mean (standard deviation), and categorical data were represented as frequencies (number of cases) and percentages as appropriate. All the study variables were compared between the two categories of IBD, namely UC and CD. Categorical data were compared between the groups using the chi-square test. A probability value (p-value) less than 0.05 was considered statistically signiﬁcant.

## Results

Our study of seven years duration from 2017 to 2024 showed that among a total of 1015 IBD patients, 752 (74.1%) had UC and 263 (25.9%) had CD. The mean age of the IBD patients was 36.6 ± 8.5 years, and that of UC and CD was 38.3 ± 8.4 and 31.8 ± 6.7 years, respectively. The distribution of the age group (Table 1) reveals that the IBD patients were more common between 31 and 40 years (53%), with UC being more common in 31 to 40 years age group (56.3%), and CD in the 21 to 30 years age group (47.5%) (p <0.001).

**Table 1:** Distribution of age group in IBD patients IBD: Inflammatory bowel disease; UC: Ulcerative colitis; CD: Crohn's disease

Age groups (years)	No. of patients with UC, n (%)	No. of patients with CD, n (%)	Total, n (%)	Chi-square value	Degrees of freedom	p-value
11-20	7 (0.9)	7 (2.7)	14 (1.4)	1578.6	6	<0.001
21-30	106 (14.1)	125 (47.5)	231 (22.8)			
31-40	423 (56.3)	115 (43.7)	538 (53)			
41-50	165 (21.9)	13 (4.9)	178 (17.5)			
51-60	32 (4,3)	0 (0)	32 (3.2)			
61-70	16 (4.3)	3 (1.1)	19 (1.9)			
71-80	3 (0.4)	0 (0)	3 (0.3)			

Among the 1015 IBD patients, 616 (60.7%) were male and 399 (39.3%) were female; among the 752 UC patients, 454 (60.4%) were male and 298 (39.6%) were female; and among the 263 CD patients, 162 (61.6%) were male and 101 (38.4%) were female. The distribution of ethnic groups of Nepal in IBD patients (Table 2) shows that IBD patients were most common (51.1%) in the Khas-Arya group, with UC being 51.9% and CD 49%; this was followed by Newars (IBD 18.8%, UC 18.9%, CD 18.6%) and then by Janajatis (IBD 18%, UC 17.3%, CD 20.2%).

**Table 2:** Distribution of ethnic groups of Nepal in IBD patients IBD: Inflammatory bowel disease; UC: Ulcerative colitis; CD: Crohn's disease

Ethnic groups	No. of patients with UC, n (%)	No. of patients with CD, n (%)	Total, n (%)	Chi-square value	Degrees of freedom	p-value
Khas-Arya	390 (51.9)	129 (49)	519 (51.1)	694.7	4	<0.001
Janajatis	130 (17.3)	53 (20.2)	83 (18)			
Newars	142 (18.9)	49 (18.6)	191 (18.8)			
Madhesi	56 (7.4)	17 (6.5)	73 (7.2)			
Others	34 (4.5)	15 (5.7)	49 (4.8)			

The distribution of UC based upon the disease location and activity (Table 3) reveals that the most common clinical phenotype of UC was left-sided (Montreal class E2: 43.8%) followed by proctitis (Montreal E1: 41.4%). Moreover, the most common occurrence of UC was of moderate activity (50.1%), followed by mild activity (39.2%). Only a few UC patients presented in severe form (10.6%).

**Table 3:** Distribution of UC based upon the disease location and activity UC: Ulcerative colitis; E1: Proctitis; E2: Left-sided disease; E3: Extensive disease

Location of UC	No. of patients with UC based upon location, n (%)	Chi-square value	Degrees of freedom	p-value	Activity of UC	No. of patients with UC based upon activity, n (%)	Chi-square value	Degrees of freedom	p-value
E1	311 (41.4)	103.9	2	<0.01	Mild	295 (39.2)	187.7	2	<0.01
E2	322 (42.8)				Moderate	377 (50.1)			
E3	119 (15.8)				Severe	80 (10.6)			

The distribution of CD based upon the disease location and activity (Table 4) shows that the most common clinical phenotype of CD was ileocolonic (Montreal class L3: 55.5%), followed by colonic involvement (Montreal class L2: 25.9%). Furthermore, the most common occurrence of CD was of moderate activity (62.4%), followed by mild activity (29.3%). Only a few CD patients presented in severe form (8.4%). There were no cases of L4 (isolated upper gastrointestinal disease); hence, L4 was omitted in the tabulated form. 

**Table 4:** Distribution of CD based upon the disease location and activity CD: Crohn's disease; L1: Terminal ileum ; L2: Colon ; L3: Ileocolon

Location of CD	No. of patients with CD based upon location, n (%)	Chi-square value	Degrees of freedom	p-value	Activity of CD	No. of patients with CD based upon activity, n (%)	Chi-square value	Degrees of freedom	p-value
L1	49 (18.6)	60.3	2	<0.01	Mild	77 (29.3)	116.9	2	<0.01
L2	68 (25.9)				Moderate	164 (62.4)			
L3	146 (55.5)				Severe	22 (8.4)			

## Discussion

Nepal has seen gradual economic growth in recent times and the corresponding rise of non-communicable diseases. Diseases such as IBD are in the stage of emergence in many developing countries like Nepal. We observed a few similarities in the clinical characteristics of IBD in our patients compared to Western patients; however, IBD in Asians could be genetically and phenotypically different from IBD in Western populations. The mean age at diagnosis of our patients was in the 30s (IBD 39.67 years, UC 38.3 years, CD 31.8 years), which was almost similar to the study from the Indian population (UC 37.5 years and CD 34.8 years) [21], and was also comparable to what was observed in some Western [22] and other Asian studies [6,8,23-25]. The age at diagnosis of our IBD patients was not very different from that of North America, Oceania, and Western Europe, where the average age at diagnosis of IBD ranged from 31 to 34 years [26], and from the Asia-Pacific region, where the median age at diagnosis of UC was 42 and that of CD was 34 [27]. However, our study had UC being more common in the 31 to 40 years age group and CD in the 21 to 30 years age group. The younger age at diagnosis in our study might reflect the changing perception of diseases in these individuals in developing nations, with the willingness to undergo investigation in the earlier stages of the diseases, and thus the overall young age of detection.

There was an overall male predominance in IBD patients in our study. This finding could be in contrast to some Western studies, which showed no gender difference. We did not observe the typical bimodal distribution of age groups that has been described in Western literature. We also studied the distribution of IBD according to various ethnic groups in Nepal. The most common race was the Khas-Arya group, followed by Newars. Ulcerative colitis was more predominant than CD in both races. Inflammatory bowel disease was found to be less prevalent in Janajatis.

We observed a few differences in the clinical phenotype of the disease as we classified the various locations of the bowels involved in UC and CD. The most common clinical phenotype of UC was left-sided disease (Montreal class E2), followed by proctitis (Montreal class E1). This finding is similar to a study from the West, where they found left-sided E2 disease more predominant in UC patients [26]. However, a few Asian studies have shown predominance of E1 disease [27,28]. There is less data on CD phenotype in Nepal. In our study, we found more ileocolonic L3 disease (55.5%), followed by colonic involvement L2 (25.9%). This is similar to the study done in the Indian population, which showed that the most common site of CD disease was ileocolonic (40.9%) [29]. Our study did not have patients with L4 disease, which could be because we did not routinely perform upper GI endoscopy in our patients unless they had symptoms. The majority of the IBD patients in our study presented in a state of moderate activity (UC 50.1% and CD 62.4%), followed by mild activity (UC 39.2% and CD 29.3%). Only a few IBD patients presented in severe form (UC 10.6% and CD 8.4%).

Our study had some limitations. This is a hospital-based study, and direct data collection was limited to patients seeking health care in our hospital. Thus, the survey was limited to symptomatic IBD patients seeking medical care. However, this is an important study of IBD in Nepal as it has a good sample size and was conducted in a center with good diagnostic facilities.

## Conclusions

With rapid urbanization and adoption of Western lifestyle, Nepal has seen a rise in IBD patients, affecting more men than women, occurring at a mean age of the 30s and mostly seen in Khas-Arya race, followed by Newars, among the Nepalese ethnic groups. A more detailed genetic analysis of this racial predisposition of IBD has to be studied in the future. We found mostly left-sided disease in UC patients, while ileocolonic disease was the most common phenotype in CD. Further large-scale, long-term, multi-center study is needed to provide more comprehensive knowledge of the clinico-epidemiological evaluation of IBD in Nepal.
